# Highly efficient methane generation from untreated microalgae biomass

**DOI:** 10.1186/s13068-017-0871-4

**Published:** 2017-07-17

**Authors:** Viktor Klassen, Olga Blifernez-Klassen, Daniel Wibberg, Anika Winkler, Jörn Kalinowski, Clemens Posten, Olaf Kruse

**Affiliations:** 1 Department of Biology/Center for Biotechnology (CeBiTec), Bielefeld University, Universitätsstrasse 27, 33615 Bielefeld, Germany; 2 Institute of Life Science Engineering (KIT), Bioprocess Engineering, University of Karlsruhe, Fritz-Haber-Weg 2, 76131 Karlsruhe, Germany

**Keywords:** Biofuel, Biogas, Methane, Microalgae mono-substrate, Nitrogen limitation, Continuous anaerobic fermentation/digestion, Maximal energy conversion efficiency, Microbial community, Ammonia/ammonium inhibition

## Abstract

**Background:**

The fact that microalgae perform very efficiently photosynthetic conversion of sunlight into chemical energy has moved them into the focus of regenerative fuel research. Especially, biogas generation via anaerobic digestion is economically attractive due to the comparably simple apparative process technology and the theoretical possibility of converting the entire algal biomass to biogas/methane. In the last 60 years, intensive research on biogas production from microalgae biomass has revealed the microalgae as a rather challenging substrate for anaerobic digestion due to its high cell wall recalcitrance and unfavorable protein content, which requires additional pretreatment and co-fermentation strategies for sufficient fermentation. However, sustainable fuel generation requires the avoidance of cost/energy intensive biomass pretreatments to achieve positive net-energy process balance.

**Results:**

Cultivation of microalgae in replete and limited nitrogen culture media conditions has led to the formation of protein-rich and low protein biomass, respectively, with the last being especially optimal for continuous fermentation. Anaerobic digestion of nitrogen limited biomass (low-N BM) was characterized by a stable process with low levels of inhibitory substances and resulted in extraordinary high biogas, and subsequently methane productivity [750 ± 15 and 462 ± 9 mL_N_ g^−1^ volatile solids (VS) day^−1^, respectively], thus corresponding to biomass-to-methane energy conversion efficiency of up to 84%. The microbial community structure within this highly efficient digester revealed a clear predominance of the phyla *Bacteroidetes* and the family *Methanosaetaceae* among the Bacteria and Archaea, respectively. The fermentation of replete nitrogen biomass (replete-N BM), on the contrary, was demonstrated to be less productive (131 ± 33 mL_N_ CH_4_ g^−1^VS day^−1^) and failed completely due to acidosis, caused through high ammonia/ammonium concentrations. The organization of the microbial community of the failed (replete-N) digester differed greatly compared to the stable low-N digester, presenting a clear shift to the phyla *Firmicutes* and *Thermotogae*, and the archaeal population shifted from acetoclastic to hydrogenotrophic methanogenesis.

**Conclusions:**

The present study underlines the importance of cultivation conditions and shows the practicability of microalgae biomass usage as mono-substrate for highly efficient continuous fermentation to methane without any pretreatment with almost maximum practically achievable energy conversion efficiency (biomass to methane).

**Graphical abstract:**
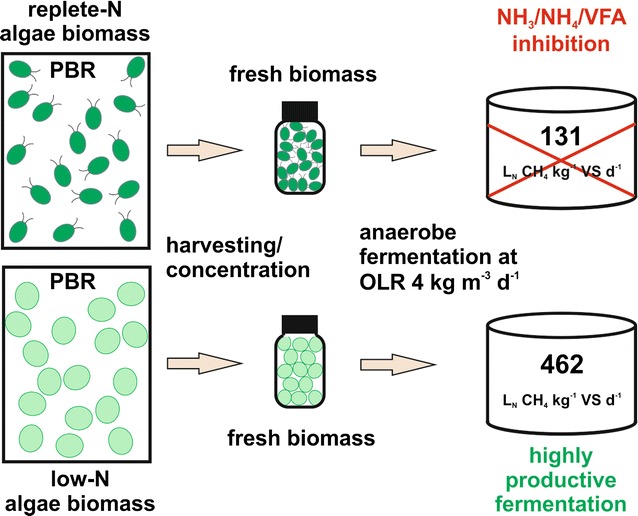
Growth condition dependence of anaerobic conversion efficiency of microalgae biomass to methane

**Electronic supplementary material:**

The online version of this article (doi:10.1186/s13068-017-0871-4) contains supplementary material, which is available to authorized users.

## Background

The steadily increasing global energy demand and limited fossil fuel sources have created tremendous efforts in developing renewable energy sources [1, 2]. Third generation biofuels, specifically derived from microalgae, are considered to be a viable alternative energy resource [3–5] because they can grow on non-arable land using fresh, saline or waste water and produce large amounts of lipids, proteins and carbohydrates over short periods of time, which can be processed into biofuels (e.g., biodiesel, bioethanol, hydrogen, methane) and valuable co-products [5–9]. Microalgae are often described as “lower” plants that never have true stems, roots, and leaves, and grow photoautotrophically by performing oxygenic photosynthesis [10], achieving biomass productivities of up to 91 tons ha^−1^ year^−1^ with relatively simple cultivation systems such as raceway ponds [11, 12]. And although mass production of microalgae is still expensive at the moment, because of their high theoretical and practical areal productivities, microalgae are in focus of research for biofuel production [3, 9, 13]. Nowadays, however, the generation of biofuels such as biodiesel or bioethanol is not economically relevant, due to the currently incurred costs for biomass production and downstream processing [13].

Methane generation via anaerobic fermentation represents an alternative way, generating gaseous fuel from biomass. Anaerobic digestion (AD) is simple in application and highly efficient, since up to 88% conversion efficiency can be reached with the appropriate substrate [14]. AD is widely used for fermentation of the so-called energy crops and organic waste material to gain methane, which is used as fuel or for electricity and heat generation [15–17]. Nevertheless, today microalgae biomass is not regarded as suitable substrate for biogas generation in AD process mainly for two reasons: (1) high recalcitrance towards microbial decomposition mediated by the rigid cell wall, and (2) unfavorable low carbon-to-nitrogen (C/N) ratio of the biomass caused by a high protein content [18, 19]. The resistance of the cell wall can be overcome by application of physical and enzymatical pretreatments [20–24], hereby unfortunately increasing investment costs for biomass processing. Additionally, the continuous fermentation of this pretreated, and thus completely accessible biomass as mono-substrate was shown to be not efficient [19], mainly due to ammonia inhibition of methanogens, released from protein degradation [20, 24–26]. To avoid the C/N imbalance of the substrate, co-fermentation with other carbon-rich substrates represents one possible strategy [27, 28]. Alternatively, some research was performed in the past, for the reduction of the protein content in the biomass by applying limited amounts of nitrogen or phosphate to the culture media [29, 30]. This strategy seem to be favoring not only lower protein content but also the accessibility of algae to microbial communities, which was monitored by methane potential tests and intact cells counting before and after the batch fermentation process [30]. Microalgae belonging to three different genera *Chlamydomonas*, *Chlorella* and *Scenedesmus* revealed with ongoing starvation status higher C/N ratios (24–26, on weight basis) in the biomass and lost subsequently the capability to resist the bacterial degradation, leading consequently to higher methane yields [30] with conversions rates near the theoretical maximum [19].

However, these experiments were performed in batch fermentation mode, allowing conclusions only regarding the accessibility of biomass towards anaerobic degradability and the achievement of maximal possible methane yields. In a regular case (industrial scale), fermentation of biomass is performed in a continuous or semi-continuous mode since this is more efficient regarding volumetric productivity. In this mode, other factors besides biodegradability can play a crucial role, e.g., ammonia or ammonium inhibition (often caused by high protein content), long chain fatty acid inhibition (caused by high lipid content), enrichment of toxic compounds and unbalance of macro/micro nutrients (necessary for growth of microbial community) [13, 15, 31]. Additionally, a variety of process parameters [hydraulic retention time (HRT), organic loading rate (OLR), temperature, pH] has to be considered for optimal performance of the digester, to avoid a complete failure of the process [19, 31].

The present study was aiming to prove the feasibility of microalgae biomass as mono-substrate, derived from nitrogen-limited growth conditions, in a long-term continuous fermentation process.

## Results and discussion

### Algae cultivation and resulting biomass properties

**Fig. 1:**
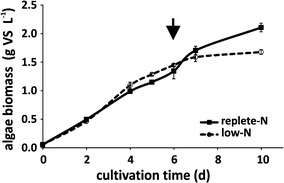
Photoautotrophic accumulation of algal biomass under replete-N and low-N culture conditions. Harvesting for fermentation experiments was performed at day 6 for both media conditions (indicated by *arrow*). *VS* volatile solids

In previous work, it was elucidated that the composition and the recalcitrance of microalgae biomass strongly depends on the growth conditions, in particular on nutrient availability and harvesting time [30]. To highlight the importance of nutrient availability, microalgae (*Chlamydomonas reinhardtii* CC-1690) biomass for the continuous fermentation was generated using cultivation media with two different nitrogen concentrations (replete-N with 11.77 mM nitrogen and low-N with 3.56 mM nitrogen, supplied as NaNO_3_). In addition, to avoid changes in biomass characteristics due to storage artifacts, e.g., freezing [32] or drying [33], algae biomass was cultured parallel to the fermentation experiments. The growth of the microalgae biomass in photobioreactors was periodically monitored by measuring organic biomass concentration (Fig. 1). According to the results from previous work [30], biomass harvesting was always performed after 6 days of cultivation for both conditions.

The phototrophic algae, cultivated in culture media with low and replete nitrogen concentrations, showed no significant differences in biomass accumulation rates at the harvesting time (Fig. 1, 6 days). After 7 days of cultivation, an obvious starvation of biomass accumulation could be monitored in low-N media, due to nitrogen depletion. In accordance with the expectation, biomass accumulation was observed in replete-N conditions up to day 10. Conclusively, no obvious disadvantages in biomass productivity (until day 6, harvesting time point) could be observed after the application of nitrogen limiting culturing conditions (Fig. 1).

The biomass composition of *C. reinhardtii* cultivated under replete-N and low-N conditions revealed significant differences regarding the protein and almost no difference in lipid content (Table 1), which is consistent with earlier observations on the total lipid and carbohydrate (mainly starch) content in *C. reinhardtii* CC-1690 under nitrogen deprivation [34, 35]. Consequently, carbohydrates represent the main carbon sink in nitrogen starved *C. reinhardtii* cells.

**Table 1:** Microalgae biomass characteristics

	Replete-N BM	Low-N BM
Proteins (% DW)	61.0 ± 5.1	28.0 ± 3.1
Carbohydrates (% DW)	21.0 ± 3.8	52.9 ± 3.5
Lipids (% DW)	20.1 ± 0.8	21.4 ± 1.2
C (% DW)	50.3 ± 1.6	46.4 ± 1.7
N (% DW)	7.3 ± 0.7	2.9 ± 0.2
Volatile solids (% DW)	95.3 ± 1.0	95.6 ± 0.4
COD (g^−g^ DW)	1.34 ± 0.11	1.31 ± 0.11
C/N ratio	6.9 ± 0.7	16.3 ± 1.1
Theoretical methane potential (mL_N_ g^−1^ VS)	~551	~549

After harvesting for fermentation, important parameters of *C. reinhardtii* biomass were determined and presented as mean values. Error bars represent standard error (SE, *n* = 8)

*BM* biomass, *DW* dry weight, *N* nitrogen, *C* carbon, *VS* volatile solids, *TMP* theoretical methane potential, *COD* chemical oxygen demand

Based on biomass composition, the theoretical methane potential was calculated using the Buswell equation [36] and empirical formula stated by Heaven et al. [37] and revealed no significant difference with approximately 551 and 549 mL_N_ g^−1^ VS between replete-N and low-N biomass, respectively (Table 1). Furthermore, corresponding to 2.2-fold lower protein content, the concentration of elemental nitrogen in the low-N biomass was decreased to only 2.9 ± 0.2% of dry weight (DW), whereas the nitrogen amount in the replete-N conditions resulted in 7.3 ± 0.7% of DW. This finding has a direct impact on the C/N ratio in the biomass, which is one of the most critical factors for a continuous fermentation process (C/N ratio: replete-N = 6.9 ± 0.7, low-N = 16.3 ± 1.1, Table 1) [38, 39]. In this particular case, the C/N ratio of the biomass, cultured under low-N conditions was within the range of 15–30, which is generally regarded as optimal for fermentation processes [15, 39, 40].

### Anaerobic digestion of microalgae biomass as mono-substrate

**Fig. 2:**
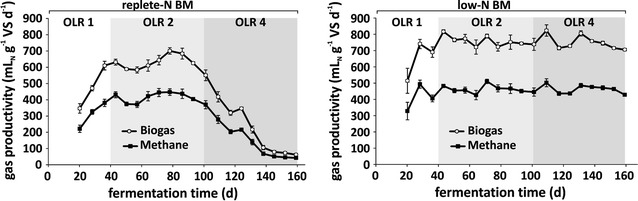
Biogas and methane productivity via anaerobic fermentation of algal biomass in continuous mode. The biogas productivity was monitored online and methane content was measured weekly (left = replete-N BM, right = low-N BM). Organic loading rate (OLR) is indicated by shades of gray in the background, thereby following biomass concentrations were applied: OLR1 = 1 g VS L^−1^ day^−1^, OLR2 = 2 g VS L^−1^ day^−1^, OLR4 = 4 g VS L^−1^ day^−1^. Error bars represent mean productivity of previous 7 days (SE, *n* = 7). *N* nitrogen, *BM* biomass, *VS* volatile solids

The continuous fermentation of algal biomass, generated under replete-N and low-N culture conditions was performed under a constant HRT of 20 days, and the organic loading rate (ORL) was subsequently increased from 1 g VS L^−1^ day^−1^ in the beginning, over 2 g VS L^−1^ day^−1^ and to 4 g VS L^−1^ day^−1^ at the end of the experiment (ORL 1, 2 and 4, respectively, Fig. 2). These loading rates were chosen since ORL 2 and 4 (2 and 4 g VS L^−1^ day^−1^, respectively) are generally used in biogas plants for continuous wet fermentation processes on industrial scale [15]. Differences in the fermentation performance of these two types of biomass were already obvious in the beginning at OLR 1 (adaptation phase), where the gas productivity was not only lower in the replete-N reactor, but was also coupled to a slower adaptation process (defined by stable biogas production). During the whole OLR 2-period, biogas as well as methane productivities were lower and less constant in the replete-N reactor compared to the low-N reactor. With the start of OLR 4, the gas productivity of the replete-N reactor started to decrease and reached the minimum level of specific biogas productivity of 62 ± 2 mL_N_ day^−1^ g^−1^ VS, at the end of the experiment. In contrast to replete-N biomass, the biogas as well as methane productivity of the low-N BM reactor remained constantly high (Fig. 2) during the whole experiment (exclusive adaptation period, OLR 1). Despite the significantly lower methane concentration in the biogas of low-N digester with 61 ± 0.4% compared to 65 ± 0.9% of replete-N digester (Additional file 1: Figure S1), the overall methane productivity was higher from low-N biomass (Fig. 2) during the complete experimental time course. The overview of the mean biogas and methane productivities, presented in Table 2, underlines that microalgae biomass from replete-N conditions can only efficiently be used at OLR 2 (2 g VS L^−1^ day^−1^). However, even this organic loading rate of replete-N biomass is already critical since the biogas productivity was not continually stable. The application of a higher loading rate (OLR 4) has a strongly negative effect on the biogas productivity from replete-N biomass (Fig. 2). On the other hand, fermentation of low-N biomass was observed to be stable over both periods OLR 2 and 4, with constantly high methane productivities of 464 ± 9 and 462 ± 9 mL_N_ g^−1^ VS day^−1^, respectively (Table 2). The overall achieved methane productivity of low-N algal biomass showed a 36% higher productivity in comparison to maize (Table 2) [41].

**Table 2:** Overview of mean biogas and methane productivities for the low-N and replete-N reactors

	Specific biogas productivity			Specific methane productivity		
	(mL_N_ g^−1^ VS day^−1^)			(mL_N_ g^−1^ VS day^−1^)		
	Replete-N BM	Low-N BM	Maize silage	Replete-N BM	Low-N BM	Maize silage
OLR 2 g VS L^−1^ day^−1^	634 ± 15	761 ± 12	740^a^	416 ± 11	464 ± 9	404^a^
OLR 4 g VS L^−1^ day^−1^	203 ± 50	750 ± 15	620^a^	131 ± 33	462 ± 9	339^a^

The values were summarized by distinct OLR-phases (OLR 2 = 2 g VS L^−1^ day^−1^, OLR 4 = 4 g VS L^−1^ day^−1^). Maize silage productivities were included for comparison as predominantly used renewable substrate for industrial scale fermentation. Error bars represent standard error (SE, *n* = 8)

*N* nitrogen, *VS* volatile solids

^a^Literature values for maize silage [41]

**Fig. 3:**
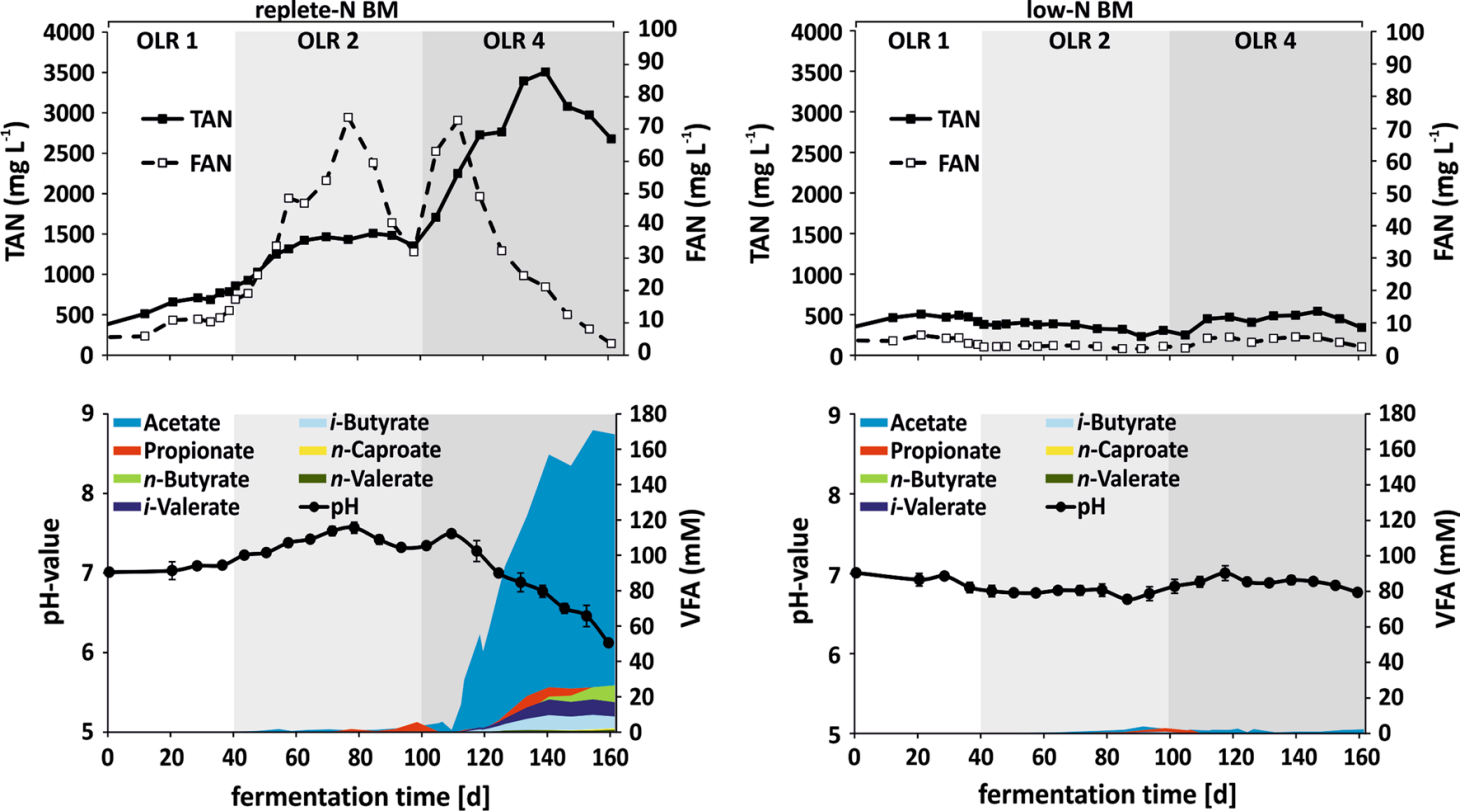
Analysis of essential fermentation parameters during anaerobic digestion of algal biomass in continuous mode. Left = replete-N BM, Right = low-N BM. Organic loading rate (OLR) is indicated by shades of gray in the background: OLR 1 = 1 g VS L^−1^ day^−1^, OLR 2 = 2 g VS L^−1^ day^−1^, OLR 4 = 4 g VS L^−1^ day^−1^. *Error bars* represent standard deviation (SD, *n* = 3). Detailed VFA concentration values in SI, Additional file 1: Table S1. N = nitrogen, *BM* biomass, *VS* volatile solids, *TAN* total ammonium nitrogen, *FAN* free ammonia nitrogen, *VFA* volatile fatty acids

Despite of the fact that the theoretical methane potential of replete-N and low-N biomass were quite similar, the specific methane productivity of low-N biomass was significantly higher compared to the biomass derived from replete-N conditions [464 ± 9 mL_N_ g^−1^ VS day^−1^ vs. 416 ± 11 mL_N_ g^−1^ VS day^−1^ at OLR 2 and 462 ± 9 mL_N_ g^−1^ VS day^−1^ vs. 131 ± 33 mL_N_ g^−1^ VS day^−1^ at OLR 4, respectively (Table 2)]. However, this finding corresponds well to previous observations, where starved biomass showed a higher accessibility and biodegradability compared to biomass from the linear growth phase [30]. To evaluate the possible reasons for the productivity differences between replete-N and low-N biomass, some essential fermentation parameters were analyzed for both reactors (Fig. 3; Additional file 1: Figures S2, S3, S4, Additional file 1: Table S1).

One of the most crucial parameters for the fermentation of protein-rich biomass is nitrogen, which is released during anaerobic decomposition of biomass in form of ammonium into the reactor supernatant [26]. Monitoring of total ammonia nitrogen (TAN) concentration in the reactor revealed indeed a huge difference between the protein-rich (replete-N BM) and low protein (low-N BM) biomass (Fig. 3). The TAN concentrations in low-N reactor were observed to be constantly below 600 mg L^−1^ during the entire experiment. However, the TAN concentration in the replete-N reactor increased at OLR 2 to a value of nearly 1500 mg L^−1^, which is already close to described inhibitory levels of 1700–1800 mg L^−1^ [26, 42, 43]. These inhibitory levels were exceeded directly after the loading rate of 4 g VS L^−1^ day^−1^ (OLR 4), reaching the maximum of 3507 ± 14 mg L^−1^ at day 140.

Nevertheless, free ammonia is known to be a more efficient inhibitor than ammonium and to have a strong negative effect primarily to the methanogens already at low concentration of 50–100 mg L^−1^ [26]. Indeed, high free ammonia nitrogen (FAN) concentration was observed in the replete-N reactor already at OLR 2 (Fig. 3), which could have had an inhibitory effect on methanogens, indicated by simultaneous decline in methane productivity at days 45–60 (Fig. 2). Yet, despite further increase of FAN to 74 ± 0.06 mg L^−1^ at day 77, the methane productivity remained stable, which may be due to Bacteria or Archaea adaptation to these FAN concentrations, and then the FAN-levels decreased again to 32 ± 0.05 mg L^−1^ (Fig. 3). At the beginning of OLR 4 (day 105), the FAN concentration in replete-N reactor increased again and reached maximal levels at day 112 with 73 ± 0.11 mg L^−1^ comparable to the maximal levels at OLR 2. Additionally, this increase was accompanied by a simultaneous increase of TAN (starting at day 105 as well), followed by a subsequent accumulation of acetate (from day 112, Fig. 3). Nevertheless, the FAN concentration started to decrease after day 112 (Fig. 3, replete-N BM, upper graph), mostly due to a drop of the pH which was caused by the constant increase of the volatile fatty acid (VFA) concentration. Especially, acetate (up to 170 mM) and other intermediate fermentation products (from day 120) such as propionate, *n*-butyrate, *i*-valerate, *i*-butyrate, *n*-caproate, *n*-valerate increased further during the time course of the experiment (Fig. 3, replete-N BM, lower graph; detailed values in SI, Additional file 1: Table S1). It can be assumed that an efficient adaptation of anaerobic microorganisms (especially methanogens) was not possible within the short time period, when the change of crucial factors such as FAN, TAN and VFA occurred. As a consequence, the process inhibition could not be surmounted, resulting in a drastic decrease of methane productivity and finally a complete failure of the fermentation process (Figs. 2, 3, replete-N BM). Similar observations were also made in other continuous fermentation approaches with protein-rich algal biomass as mono-substrate, where high TAN/FAN concentrations, and consequently increasing VFAs have led to decreased methane productivities [20, 24, 25, 29, 44, 45].

On the other hand, the reactor, fed with low-N biomass, did not show any imbalances in fermentation parameters, being constantly low throughout the entire experiment (Fig. 3, low-N BM). Especially, the FAN concentration showed values lower than 5 mg L^−1^ during the complete experimental time, far below inhibitory levels [26]. Furthermore, this observation is also reflected by constantly high methane productivity at different loading rates (Fig. 2, low-N BM, Table 2).

Since the fermentation of microalgae biomass, generated under nitrogen limited conditions was stable and produced constant amounts of methane, it was interesting to evaluate the conversion efficiency level of this process. For this purpose, the theoretical methane potential (TMP) of the biomass was compared to the specific methane productivity reached in the experiments [46]. According to our calculations, the conversion efficiency for low-N biomass to methane reached 84% [calculation specific methane productivity (Table 2) of TMP (Table 1)] for both loading rates (OLR 2 and 4). Having in mind that approximately 12–15% of the organic matter is used for bacterial growth and maintenance requirements during the anaerobic digestion process [14], and therefore being not available for fermentation to methane. The fermentation of low-N biomass within this study reached almost the maximal capacity and represents the most efficient process so far described in the literature for algal biomass as a mono-substrate [19]. For instance, Samson and colleagues observed maximal methane productivity by digestion of *Spirulina maxima* of only 350 mL_N_ g^−1^ VS day^−1^, and thus a maximal conversion efficiency of 59%. These results, however, were achieved only under OLR 1 and HRT of 30 days, whereas the productivities decreased significantly when higher loading rates were applied, due to pronounced ammonia inhibition [45]. Even lower maximal productivities of only 267 mL_N_ CH_4_ g^−1^ VS day^−1^ (at OLR 4 and HRT of 20 days) were obtained in another recent study using *Spirulina* biomass [47]. Similar results could be achieved for green algae biomass in other studies, where only 160 mL_N_ CH_4_ g^−1^ VS day^−1^ could be reached for raw *Chlorella vulgaris* biomass, corresponding to 32% conversion efficiency. After thermal pretreatment of the biomass, the yield could be increased by 1.5-fold and still reached only 233 mL_N_ CH_4_ day^−1^ g^−1^ VS corresponding to only 49% of TMP (OLR 0.8, HRT 15) [25]. Very low methane productivities of only 70 mL_N_ day^−1^ g^−1^ VS were published by Mahdy and co-workers for *C. vulgaris*, corresponding to only 15% conversion efficiency (OLR 1, HRT 15). Nevertheless, parallel digestion of enzymatically pretreated algae biomass was 2.2 times more efficiently digested and resulted in 196 mL_N_ CH_4_ day^−1^ g^−1^ VS corresponding again to only 49% of TMP (OLR 1, HRT 20) [20]. Moreover, in comparison to the fermentation performance with microalgae, the theoretical maximum achieved for macroalgae substrate was in the range of 25–45% [48]. Moreover, the methane productivity from macroalgae fermentation lies often in the range of less than 300 mL CH_4_ g^−1^ VS day^−1^ [27, 49, 50], which is significantly lower compared to the productivity of 462 mL_N_ CH_4_ g^−1^ VS day^−1^ achieved in this work with microalgae. Apart from the finding that the methane yield from batch experiments with macroalgae biomass [27] is rather low compared to microalgae, the continuous fermentation under comparable conditions (regarding loading rate) seems also to be less efficient and sensible towards residual salt content in the biomass due to marine origin [49].

Thus, the biomass-to-methane conversion efficiency of 84% demonstrated within this work by the application of low-N algae biomass is not only significantly higher compared to other long-term fermentation trails with untreated biomass but also compared to the results achieved after successful pretreatment of microalgae biomass. Furthermore, this efficiency may represent the maximum practically achievable under the AD conditions [14]. Considering the energy consumption of microbial biomass, the practical efficiency of the fermentation process presented here is at 96–99%, and thus the process may be described as optimal. Based on these “proof of concept” results, this strategy can also be performed under more applied levels. So for instance, the cultivation of microalgae under non-axenic conditions was tested and revealed rather low/negligible contamination levels due to the nature of the photoautotropic culture media (especially low-N conditions) and no negative effect during the fermentation process of this biomass could be observed (unpublished observations). Additionally, other more industrially relevant microalgae species can also be tested in continuous fermentation mode, since our previous batch results for *Parachlorella kessleri* and *Scenedesmus obliquus* were quite promising, exhibiting very similar properties in terms of C/N ratios and methane yields such as *C. reinhardtii* [30]. Moreover, to reduce the cultivations costs of the microalgae and to include more positive environmental aspects to the process, wastewater could be used as nutrition source and flue gas (e.g., biogas after combustion) could be integrated as CO_2_ source in the process [27].

### Consequence of the fermentation parameter on the microbial community

**Fig. 4:**
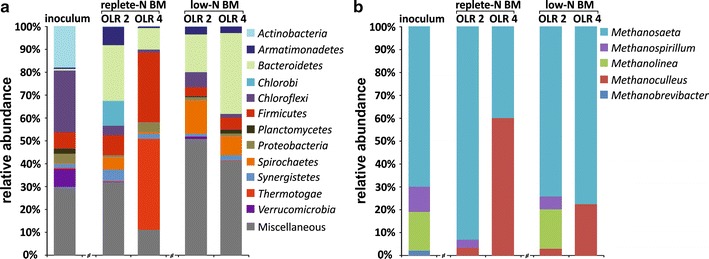
Bacterial diversity dynamic as assessed by high-throughput 16S rDNA amplicon sequencing. The data is represented at the phyla level for Bacteria (**a**) and family level for Archaea (**b**). The reactors fed with biomass cultivated with replete and low nitrogen content (replete-N BM and low-N BM) were exposed to increasing organic loading rates OLR 2 (2 g VS L^−1^ day^−1^) and OLR 4 (4 g VS L^−1^ day^−1^). The inoculum and the sampling periods at the end of each OLR were chosen for microbial community monitoring

High-throughput 16S rRNA gene amplicon sequencing was accomplished to investigate how this suboptimal and optimal performance of the replete-N and low-N biomass digesters is reflected on the microbial community. For the comparison of the dynamics of the bacterial community in the different conditions, samples of inoculum (local waste water treatment plant) and the biogas fermenter, fed with replete-N and low-N biomass on the end of OLR 2 (after 100 days) and OLR 4 (after 160 days) were chosen. In all investigated samples, no evidence of eukaryotic plastid 16S rRNA could be found, suggesting that the algal DNA was completely disintegrated during the anaerobic fermentation. Based on the 16S rRNA gene amplicon database (RDP) [51], the biogas producing microbial community was dominated by Bacteria with 99%, and the Archaea was only represented with approximately 1% (Fig. 4). These findings have previously been reported [52–55], and are in agreement with the fact that bacteria are involved in the first three steps of biomass transformation with a high variety of substrate preferences, and Archaea are restricted to a very narrow substrate spectrum in terms of acetate, methyl-group containing compounds as well as CO_2_ and H_2_.

According to *The prokaryots* [56] and *Bergey’s Manual of Systematic Bacteriology* [57, 58], all identified bacterial community members within the investigated samples are typically involved in the anaerobic degradation of the supplied feedstock as they are described to have cellulolytic, saccharolytic, glycolytic, lipolytic, proteolytic and/or acido-/acetogenic capacities. However, many of the bacterial 16S rRNA amplicon reads (27.26 ± 2.75% for inoculum, 28.94 ± 1.37 and 10.39 ± 0.43% for replete-N BM OLR 2 and OLR 4, as well as 48.01 ± 1.77 and 40.58 ± 1.59% for low-N BM OLR 2 and OLR 4, respectively, Fig. 4a) could not be classified at the phylum levels, respectively, confirming that largely bacterial communities in AD reactors remain unknown [59].

The active sludge (inoculum) revealed very high species diversity comprised 603 ± 52 OTUs (Additional file 1: Table S2). Overall, 73% of the identified sequence reads could be assigned to 18 different phyla, with the most abundant among them the members of the phyla *Chloroflexi* (26.78%), *Actinobacteria* (17.96%), *Verrucomicrobia* (7.80%) and *Firmicutes* (7.01%), whereas all other phyla were found only to a minor portion (Fig. 4a). The bacterial diversity dropped significantly during the anaerobic fermentation of algal biomass as mono-substrate, cultivated under replete-N and low-N culture conditions and revealed 178 ± 34 and 111 ± 7 OTUs, as well as 269 ± 20 and 177 ± 2 OTUs for OLR2 and OLR4, respectively (Additional file 1: Table S2). This development indicates that distinct bacteria species begun to dominate due to the selection pressure based on the certain substrate type and amount and other species were extinct. Similar observations were obtained in other studies [60, 61].

Furthermore, in the reactors with no obvious inhibition, the members of the phyla *Bacteroidetes* became dominant in the AD process, followed by *Chlorobi* in the digester with replete-N biomass at OLR 2 or *Spirochaetes* with low-N biomass at OLR 2 and 4 (Fig. 4a). Interestingly, within the phylum *Bacteroidetes*, mainly three different main OTUs were identified (OTU_2, 3 and 26; Additional file 1: Figure S5). OTU_26 is representing the genus *Paludibacter* of the family *Porphyromonadaceae,* which was described to ferment various sugars to acetate and propionate as the major fermentation products [58], and is mostly abundant in the low-N BM digester with high amount of carbohydrates (Table 1). The phyla *Chlorobi* is represented by only one member of the genus *Ignavibacterium* (OTU_36, Additional file 1: Figure S5), which was also described to utilize various carbohydrates [56]. The phyla *Spirochaetes* mainly consists of two OTUs of the order *Spirochaetales* (OTU_8 and 18, Additional file 1: Figure S4), of which OTU_18 could be classified to the genus *Treponema* that utilizes carbohydrates and/or amino acids as carbon and energy source [58]. Interestingly, the digester (replete-N BM, OLR 4), which experienced acidosis because of the high FAN/TAN and VFA concentrations (Fig. 3 replete-N BM), showed a completely different bacterial population, with the members of phyla *Firmicutes* and *Thermotogae* being the most abundant in this samples (Fig. 4a). Thereby, the *Firmicutes* were to 70% represented by the genus *Sporanaerobacter* (OTU_108), and the *Thermotogae* to 99.9% by the species (OTU_125, Additional file 1: Figure S5) similar to *Defluviitoga tunisiensis* [62]. *Sporanaerobacter* was described to be able to utilize some sugars, peptides and various single amino acids into acetate [57, 63]. Moreover, members of *Thermotogae* have been characterized for complex polysaccharide fermentation and hydrogen production [62, 64], what might promote beneficial associations with hydrogenotrophic methanogens [65]. The phyla *Bacteroidetes* is also present in these samples, however, it is in contrast to the well-performing digesters, mainly represented by other members of the family *Porphyromonadaceae* (OTU_78 and 111, Additional file 1: Figure S5). The most members of the family *Porphyromonadaceae* are primarily described to be weakly saccharolytic in contrast to *Paludibacter* observed in well-performing digester, since the bacterial growth was not observed to be significantly affected by carbohydrates, but is enhanced by protein hydrolysates [58], which is also in agreement with the fact that this digester was fed with protein-rich biomass.

In general, archaeal communities were much less diverse than bacterial ones (Fig. 4a, b), with *Methanomicrobiaceae*, *Methanobacteriaceae* and *Methanosaetaceae* being the dominant families. The members of *Euryarchaeota* in the inoculum (active sludge of the local waste water treatment plant) are present to 1.18% ± 0.13 and are consistent on the genus level of *Methanobrevibacter*, *Methanolinea* and *Methanospirillum* and *Methanosaeta*, with the last being the most abundant of the methanogenic community. This finding is also in agreement with the general consideration of the acetoclastic activity being the dominant methanogenic pathway [66, 67]. Distribution, similar to the inoculum, could be observed in the well-performing (replete-N BM OLR 2 and low-N BM OLR 2, 4) digesters, with *Methanosaeta* sp. representing the most abundant Archaea in the methanogenic community, followed by *Methanoculleus* sp. and *Methanospirillum* sp. and *Methanolinea* sp (Fig. 4b). On the other hand, the archaeal community in replete-N BM digester OLR 4 is dominated by *Methanoculleus* sp. and to lesser extent by *Methanosaeta* sp., suggesting an apparent redirection from the acetoclastic towards hydrogenotrophic methanogenesis. The increased abundance of *Methanoculleus* sp. could possibly be attributable to the sensitivity of acetoclastic Archaea towards volatile fatty acid intoxication (acidosis) and/or higher availability of hydrogen provided by certain bacterial species [68] like the members of the phyla *Thermotogae*. Similar behavior could be also observed in other studies, whereby the authors suggested that the replacement of the dominant *Methanosaeta* sp. by *Methanoculleus* sp. might be a potential warning indicator of acidosis within the fermenter [60, 61, 69].

## Conclusions

Biogas generation from microalgae biomass has been researched for approximately 60 years with the major outcome that microalgae represent a rather challenging substrate for anaerobic digestion due to high cell wall recalcitrance and unfavorable C/N ratio, owing to its high protein content [18, 19]. The present study investigated the anaerobic digestion from microalgae biomass generated in replete-N as well as naturally occurring (nitrogen limitation, low-N) conditions. The use of algal biomass from replete nitrogen conditions, especially at OLR 4 have led to an inhibition of the digester, caused by high TAN/FAN and VFA concentrations, and thus to fermentation failure with very low methane productivity. In the failed reactor (replete-N biomass, OLR 4), a clear shift could be observed in the bacterial community to the phyla *Firmicutes* and *Thermotogae* and archaeal population changed from acetoclastic to hydrogenotrophic methanogenesis.

In contrast to fermentation of replete-N biomass, the application of nitrogen limitation during the microalgae cultivation resulted in generation of biomass with significant changes in the composition (highly accessible biomass with two times lower protein content), and thus in an optimal mono-substrate for efficient AD process in continuous manner. The fermentation process was characterized by stable process parameters with very low levels of main inhibitory compounds. The investigation of the microbial communities revealed *Bacteroidetes* phyla as subsequently dominating the efficiently preforming digester, indicating that these members adapted most efficiently to the microalgae mono-substrate. Furthermore, among the methanogens, the family of *Methanosaeta* sp. was predominant, suggesting the acetoclastic methanogenesis to be the main pathway during the successful anaerobic degradation of microalgae. The productivity of methane was constantly on a high level (464 ± 9 and 462 ± 9 mL_N_ g^−1^ VS day^−1^ at OLR of 2 and 4, respectively), thus corresponding to an energy conversion efficiency (biomass to methane) of 84%. Taken into account the amount of organic matter used to form new microbial cells and energy for cell metabolism was 12–15% [14], algae substrate conversion efficiency reached in this study almost the practically achievable maximum of 96–99%. According to these considerations, algae biomass can be used highly efficiently for AD without any energy or cost intensive pretreatments. Thus, the presented results of the efficient continuous fermentation of low-N biomass are moving the industrial application of biofuel generation from algal biomass in a more economically feasible direction, especially because the generation of algae biomass under these conditions saves significantly expensive fertilizers (e.g., nitrogen).

## Methods

### Strains and growth conditions

*Chlamydomonas reinhardtii* strain CC-1690 from the Chlamydomonas Center (Duke University, Durham NC, USA) was used for all experiments. Liquid algal cultures were grown photoautotrophically under continuous white light (300 µmol photons m^−2^ s^−1^; Osram L 36 W/865, Osram Germany). Cultivations were conducted in glass bottles (DURAN^®^, max. capacity 3.5 L, outer diameter 110 mm and 450 mm high, Schott Germany) with 3 L of algae culture, under continuous agitation on a magnetic stirrer. Carbon supply was achieved by bubbling with moisture pre-saturated, carbon dioxide-enriched air (3% v/v) with a flow rate of 5 L h^−1^. Nutrients were provided by a modified Provasoli based minimal medium [70]. For replete nitrogen culture conditions, the following components and concentrations were applied: K_2_HPO_4_ 0.57 mM; H_3_BO_3_ 0.16 mM; MgSO_4_ 4.87 mM; KCl 21.46 mM; NaNO_3_ 11.77 mM; CaCl_2_ * 2H_2_O 2.72 mM; FeCl_3_ * 6 H_2_O 12.2 µM; Na_2_-EDTA 12.5 µM; EDTA 103 µM; ZnCl_2_ 2.2 µM; MnCl_2_ * 4H_2_0 16.7 µM; CoCl_2_ * 6 H_2_O 50.4 nM; CuCl_2_ * 2H_2_O 17.6 nM; Na_2_MoO_4_-* 2H_2_O 24.8 nM. Low-nitrogen cultivation conditions were realized according to previous work [30] by applying a limited amount of nitrogen (3.56 mM NaNO_3_ equals to 50 mg of nitrogen per liter culture).

### Determination of algal biomass concentration

The biomass concentration was determined by centrifugation of 15 mL of cell culture (3000×*g* for 5 min, at least three technical replicates per sample) and drying of the cell pellet in a pre-weighted glass tube at 105 °C for 24 h. To determine the organic biomass fraction, the sample tubes were subsequently incubated at 550 °C for 5 h and the residual ash determined by weighing. The amount of organic biomass (dry weight minus the ash content) was calculated and expressed as volatile solids (VS, g L^−1^).

### Measurement of elemental N and C content in the biomass (C/N ratio)

Total carbon (C) and nitrogen (N) content of the algal biomass was determined via an element analyzer (VARIO EL III, Elementar Analysesysteme, Hanau, Germany) as described before [71].

### Anaerobic fermentation and quantitative biogas measurement

The continuous fermentation of algae biomass was performed according to the VDI 4630 guideline [46]. Fermentation was performed in B Braun glass fermenters, maximal capacity of 2 L. Fermentation temperature of 38 °C was provided by external tempered water bath via water circulation thought a build-in water jacket in the fermenter. Reactor content was stirred at 100 rpm via slices stirring system (in a 15 min. ON- and 15 min OFF-mode). The digester was operated with 1 L working volume (inoculated with microbial community from anaerobic digester of a local waste water treatment plant Bielefeld–Heepen, Germany) and constant hydraulic retention time (HRT) of 20 days. Feeding/withdrawing was performed manually with a syringe (first 50 mL rector content out, thereafter 50 mL algae substrate in) daily (despite of semi-continuous feeding mode, the fermentation is designated here, in agreement to VDI guideline [46] as a continuous process). Biogas (water free after condensations column) evolution was measured continuously by a MilliGascounter^®^ (MGC-1 V 3.0, 3, 2 mL, Ritter, Germany) and evaluated by applying RIGAMO Software (Ritter, Germany), followed by normalization of the gas volume to standard temperature of 0 °C. Organic loading rate (OLR) was increased subsequently and simultaneously in both digesters [fed with replete-N and low-N biomass (BM)], from day 0 to 40 (OLR 1 = 1 g VS L^−1^ day^−1^), from day 40 to 100 (OLR 2 = 2 g VS L^−1^ day^−1^) and from day 100 to 160 (OLR 4 = 4 g VS L^−1^ day^−1^). The fresh algal substrate was obtained by centrifugation of the cultures at 3000×*g* for 5 min and removal of the supernatant. To avoid freezing or drying artifacts, biomass was diluted by addition of H_2_O to required concentration and stored by 2 °C prior feed (max. 2 weeks long).

### Methane content measurement via gas chromatography (GC)

The determination of the methane content within the biogas was performed by GC analysis weekly in nine technical replicates. Biogas from the fermenter was sampled with a gas tight syringe (5 mL) through a rubber seal and injected into a gas chromatograph GCM MicroBox III (Elster GmbH, Germany) equipped with an Micropacked HayeSep A-Column (Length: 65 cm, inner diameter: 0.3 mm) and a thermal conductivity detector (TCD). Column temperature in the first 50 s was at 50 °C with following linear increase 4 °C s^−1^ to 165 °C witch was hold constant till the end by 120 s. Argon was used as the carrier gas and the calibration of the GC was performed with defined gas (Linde, Germany) containing O_2_(0.103%), H_2_S(0.208%), H_2_(0.498%), CH_4_(59.4%), CO_2_(34.4%) and N_2_(5.391%), mixed according to DIN EN ISO 6141.3.

### Determination of biomass composition and theoretical methane potential

Determination of lipid fraction was performed in two technical and four biological replicates from 50 mg of lyophilized biomass each. After homogenization (3 × 30 s at 6500 rpm using a Precellys 24, Peqlab, Erlangen, Germany), the total lipid fraction was extracted according to a modified Folch protocol [72] using a total of 4 mL of methanol and 8 mL of chloroform. Contaminants were removed by washing the extract with 3 mL of deionized water. After evaporation of solvents under nitrogen atmosphere, lipid fraction was determined via gravimetrical measurement. The total cellular protein amount was determined using Bio-Rad DC Protein assay (Bio-Rad, CA, USA). The amount of total carbohydrates was determined using the protocol according to Dubios et al. [73]. The theoretical methane potential was calculated in accordance with Buswell equation and empirical formula stated by Heaven et al. with TMP`s of 446, 415 and 1014 mL_N_ g^−1^ VS for proteins, carbohydrates and lipids, respectively. Within the formula *P* stays for protein, *C* for carbohydrate and *L* for lipid content on VS basis.

$$\begin{aligned}{\text{TMP}}\left( {{\text{mL}}_{{\text{N}}} {\text{g}}^{{ - 1}} {\text{VS}}} \right)& = \frac{{P\% }}{{\sum {\left( {P\% + C\% + L\% } \right)} }} \times 446 \\ & \quad + \frac{{C\% }}{{\sum {\left( {P\% + C\% + L\% } \right)} }} \times 415 \\ & \quad + \frac{{L\% }}{{\sum {\left( {P\% + C\% + L\% } \right)} }} \times 1014 \hfill \\ \end{aligned}.$$

### Determination of the fermentation parameters

Total ammonium nitrogen (TAN) was determined using colorimetric verification via cuvette tests LCK302 (Hach Lange GmbH, Germany). Free ammonia nitrogen (FAN) was calculated from TAN value in respect to temperature and pH according to the formula given by Astals and colleagues [74]. Total organic- and inorganic-carbon (TOC and TIC, respectively) were measured via LCK381, total nitrogen was determined via LCK 338, (Hach Lange GmbH, Germany). The determination of volatile fatty acid (VFA) concentrations was performed via GC-FID analysis. Sample preparation was done according to the 5560D procedure [75] and analyzed using a Shimadzu GC-2010 plus Gas Chromatograph equipped with a Macherey–Nagel OPTIMA^®^ FFAPplus (Length: 30 m, inner diameter: 0.25 mm) column (Macherey–Nagel, Germany) and coupled to an FID detector (supplied with H_2_ and synthetic air). Analysis was performed under constant pressure of 231.9 kPa with He as carrier gas and N_2_ as makeup gas with constant flow rate of 60 cm s^−1^. Column temperature in the first 2 min was at 100 °C with following linear increase to 175 °C within 15 min. VFA-Mix standard (46975-U, Supelco Analytical, Sigma-Aldrich, USA) at concentrations of 0.1, 1 and 10 mM was used for calibration.

### Microbial monitoring by high-throughput 16S rRNA amplicon sequencing

Genomic DNA was extracted as previously described by Zhou et al. [76]. For the determination of the taxonomic profile of the biogas community, high-throughput sequencing of the hypervariable V3–V4 regions of the 16S rRNA gene was performed on the Illumina MiSeq system by applying the paired-end protocol, according to the manufacturer’s instructions and using of the Illumina recommended gene specific primer sequences [77]. For the data processing and analysis, an amplicon analysis pipeline was used as recently described [59, 78]. Briefly, raw sequences were merged by FLASH [79] and further processed and analyzed using the UPARSE pipeline [80] based on Usearch 8.0 [81] with default settings. Processed operational taxonomic units (OTU) were taxonomically classified using the RDP classifier 2.7 [51].

## Additional file

**Additional file 1: Figure S1.** Methane concentration in the biogas, produced during the fermentation of replete-N and low-N algae biomass (replete-N BM and low-N BM, respectlively). Statistics: two-sample t-test with 95% confidence interval. **Figure S2.** Concentration of total carbon and nitrogen during the experimental time course in replete-N BM digester (A) and low-N BM digester (B). Concentration of total organic and inorganic carbon (TOC and TIC) is shown for replete-N BM digester (C) and low-N BM digester (D). Measurements were performed in three replicates; error bars represent standard deviation (SD). **Figure S3.** Concentration of volatile and total solids (VS and TS, respectively) during the experimental time course in replete-N BM digester (A) and low-N BM digester (B). Measurements were performed in at least three replicates; error bars represent standard deviation (SD). **Figure S4.** Concentration of chemical oxygen demand (COD) during the experimental time course in replete-N BM digester (A) and low-N BM digester (B). Measurements were performed in three technical replicates; error bars represent standard deviation (SD). **Figure S5.** Bacterial diversity dynamics as assessed by high-throughput 16S rRNA amplicon sequencing and represented at the OTU level. The reactors, fed with biomass cultivated in media with replete and low nitrogen content (replete-N BM and low-N BM) were exposed to increasing organic loading rates of 2 and 4 g VS L^-1^ d^-1^ (OLR 2 and OLR 4, respectively). The inoculum and the sampling periods at the end of each OLR were chosen for microbial community monitoring. **Table S1.** Analysis of the volatile fatty acid (VFA) content during the time course of the experiment. The identification and quantification of the intermediate fermentation products (mM) was determined via GC-FID. The indicated error (±) represents standard deviation (SD, n = 2). **Table S2.** Filtered sequences during amplicon processing. OTU=operational taxonomic unit, N=nitrogen, sd=standard deviation, OLR=organic loading rate, rep=replicate.

## Abbreviations

AD: anaerobic digestion
C/N: carbon-to-nitrogen ratio
DW: dry weight
BM: biomass
SE: standard error
SD: standard deviation
FAN: free ammonia nitrogen
TAN: total ammonium nitrogen
OLR: organic loading rate
HRT: hydraulic retention time
VFA: volatile fatty acids
C: carbon
N: nitrogen
TMP: theoretical methane potential
VS: volatile solids
