# Supplementary material for: Highly efficient methane generation from untreated microalgae biomass
Source: Biotechnol Biofuels. 2017 Jul 17;10:186. doi: 10.1186/s13068-017-0871-4 (PMC5513056; doi:10.1186/s13068-017-0871-4)
Supplement: Supplementary file 1 — Additional file 1: Figure S1. Methane concentration in the biogas, produced during the fermentation of replete-N and low-N algae biomass (replete-N BM and low-N BM, respectlively). Statistics: two-sample t-test with 95% confidence interval. Figure S2. Concentration of total carbon and nitrogen during the experimental time course in replete-N BM digester (A) and low-N BM digester (B). Concentration of total organic and inorganic carbon (TOC and TIC) is shown for replete-N BM digester (C) and low-N BM digester (D). Measurements were performed in three replicates; error bars represent standard deviation (SD). Figure S3. Concentration of volatile and total solids (VS and TS, respectively) during the experimental time course in replete-N BM digester (A) and low-N BM digester (B). Measurements were performed in at least three replicates; error bars represent standard deviation (SD). Figure S4. Concentration of chemical oxygen demand (COD) during the experimental time course in replete-N BM digester (A) and low-N BM digester (B). Measurements were performed in three technical replicates; error bars represent standard deviation (SD). Figure S5. Bacterial diversity dynamics as assessed by high-throughput 16S rRNA amplicon sequencing and represented at the OTU level. The reactors, fed with biomass cultivated in media with replete and low nitrogen content (replete-N BM and low-N BM) were exposed to increasing organic loading rates of 2 and 4 g VS L-1 d-1 (OLR 2 and OLR 4, respectively). The inoculum and the sampling periods at the end of each OLR were chosen for microbial community monitoring. Table S1. Analysis of the volatile fatty acid (VFA) content during the time course of the experiment. The identification and quantification of the intermediate fermentation products (mM) was determined via GC-FID. The indicated error (±) represents standard deviation (SD, n = 2). Table S2. Filtered sequences during amplicon processing. OTU=operational taxonomic unit, N=nitrogen, sd=stand [file 13068_2017_871_MOESM1_ESM.docx]

**Supplementary information**

**Highly efficient methane generation from untreated microalgae biomass**

Viktor Klassen^1^, Olga Blifernez-Klassen^1^, Daniel Wibberg^1^, Anika Winkler^1^, Jörn Kalinowski^1^, Clemens Posten^2^, Olaf Kruse^1^*

^1^ Bielefeld University, Department of Biology/Center for Biotechnology (CeBiTec), Universitätsstrasse 27, 33615 Bielefeld, Germany

^2^ University of Karlsruhe, Institute of Life Science Engineering (KIT), Bioprocess Engineering, Fritz-Haber-Weg 2, 76131 Karlsruhe, Germany

**Corresponding author**: [olaf.kruse@uni-bielefeld.de](mailto:olaf.kruse@uni-bielefeld.de), Tel: +49-(0)521-106-12258, Fax: +49-(0)521-106-12290


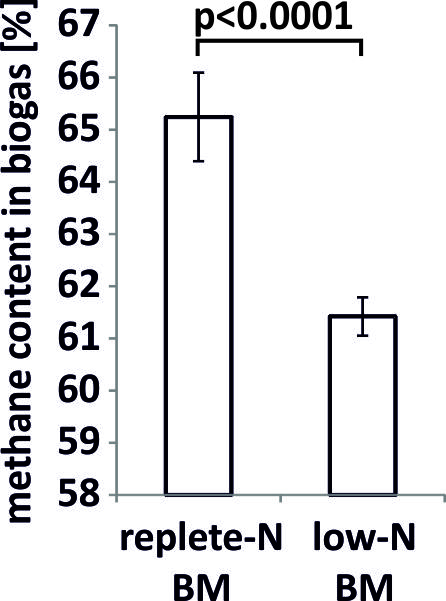


Figure S1: Methane concentration in the biogas, produced during the fermentation of replete-N and low-N algae biomass (replete-N BM and low-N BM, respectlively). Statistics: two-sample t-test with 95% confidence interval.


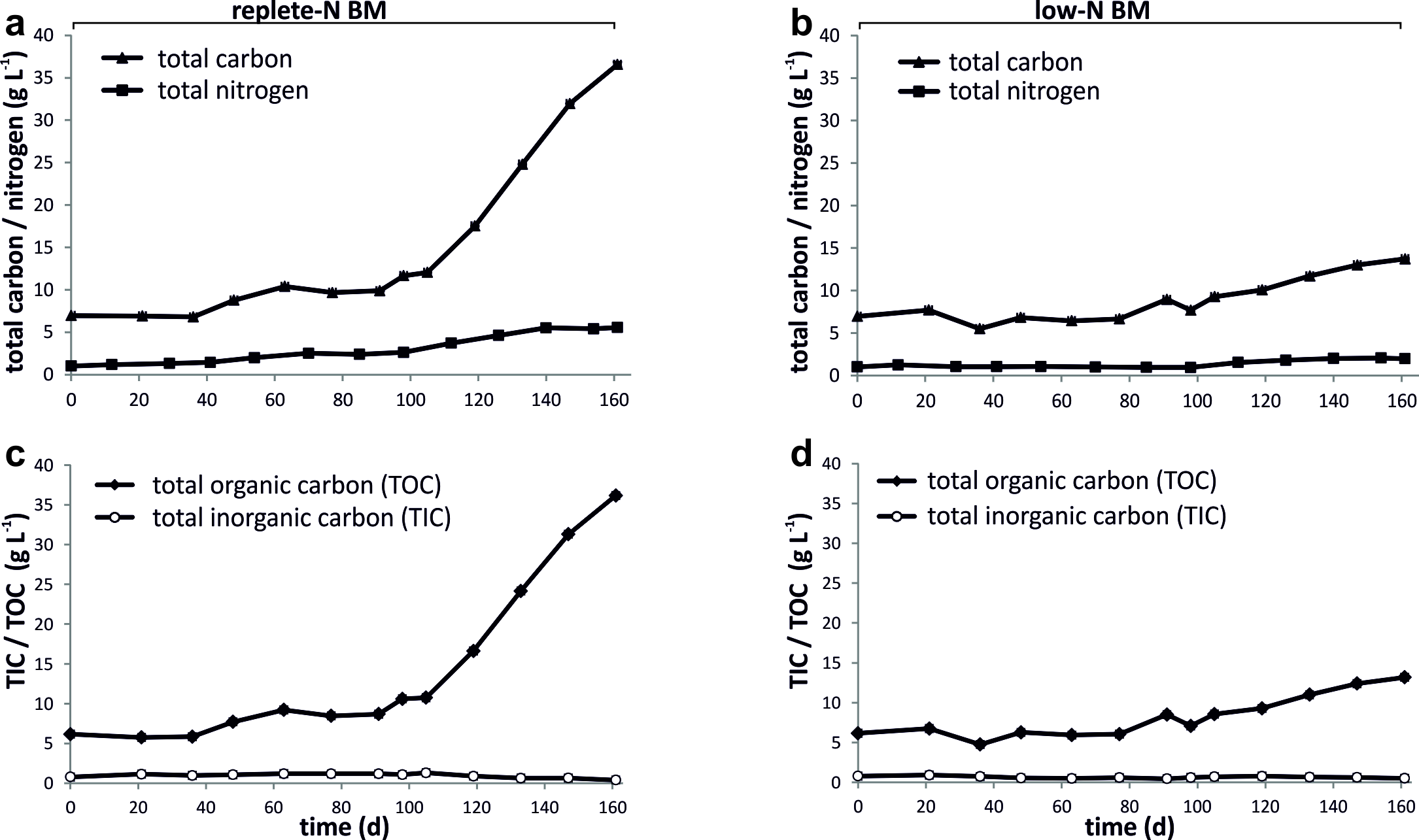


Figure S2: Concentration of total carbon and nitrogen during the experimental time course in replete-N BM digester (A) and low-N BM digester (B). Concentration of total organic and inorganic carbon (TOC and TIC) is shown for replete-N BM digester (C) and low-N BM digester (D). Measurements were performed in three replicates; error bars represent standard deviation (SD).


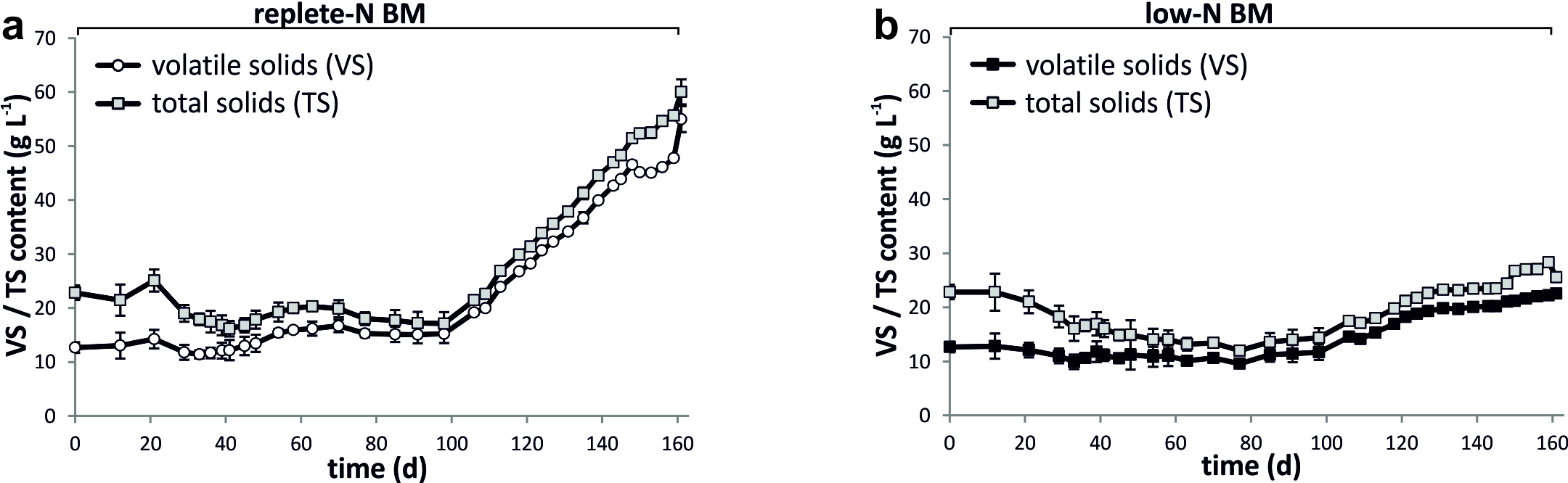


Figure S3: Concentration of volatile and total solids (VS and TS, respectively) during the experimental time course in replete-N BM digester (A) and low-N BM digester (B). Measurements were performed in at least three replicates; error bars represent standard deviation (SD).


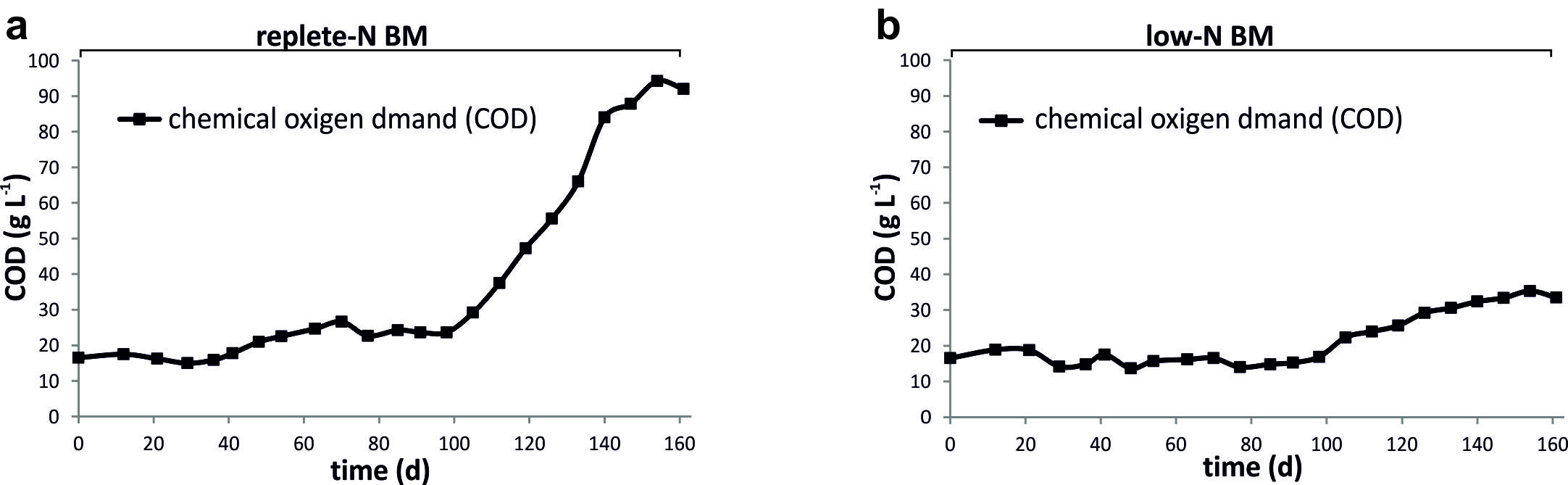


Figure S4: Concentration of chemical oxygen demand (COD) during the experimental time course in replete-N BM digester (A) and low-N BM digester (B). Measurements were performed in three technical replicates; error bars represent standard deviation (SD).

**Table S1**: Analysis of the volatile fatty acid (VFA) content during the time course of the experiment. The identification and quantification of the intermediate fermentation products (mM) was determined via GC-FID. The indicated error (±) represents standard deviation (SD, n = 2).

**Table S2**: Filtered sequences during amplicon processing. OTU=operational taxonomic unit, N=nitrogen, sd=standard deviation, OLR=organic loading rate, rep=replicate

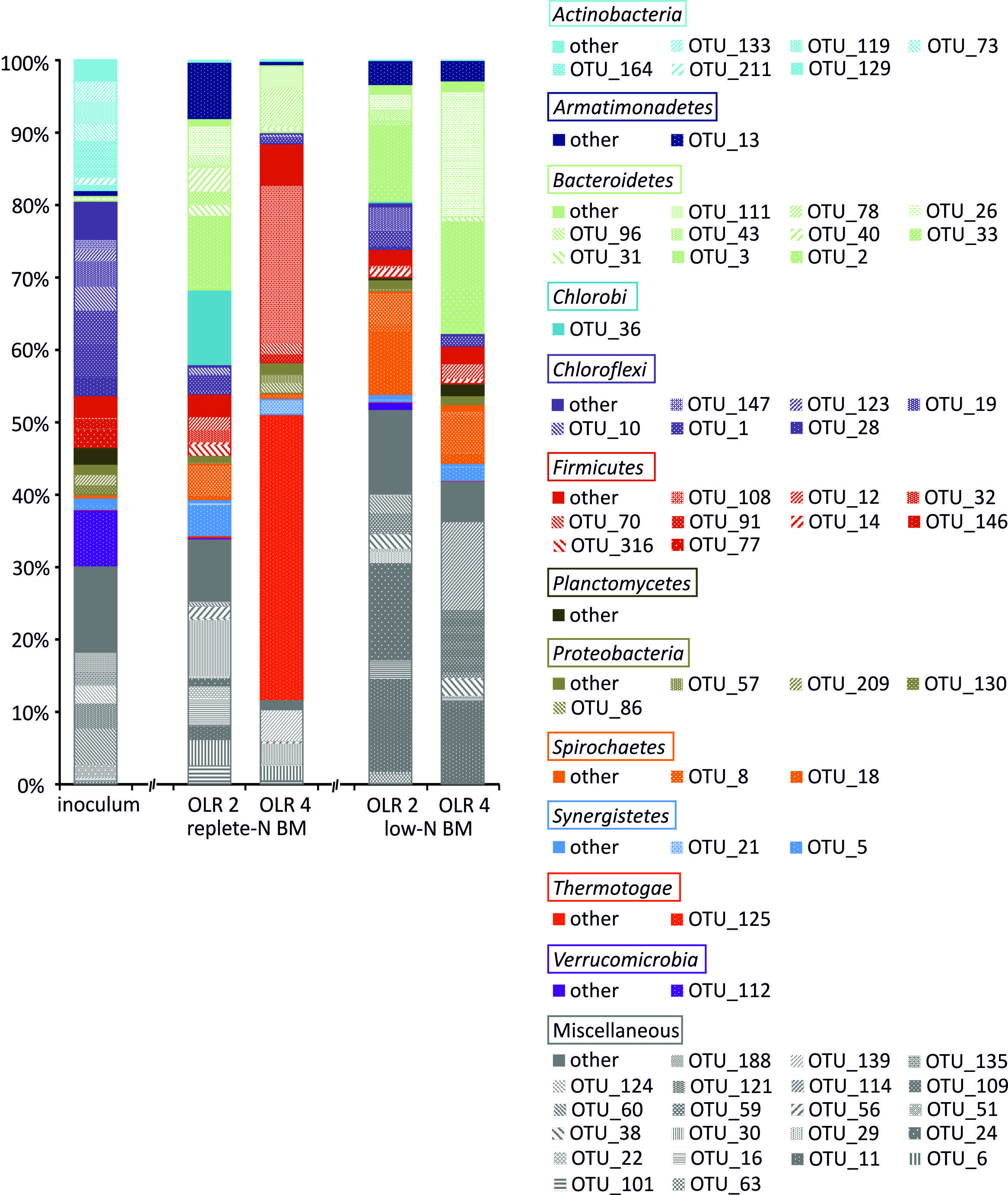


Figure S5: Bacterial diversity dynamics as assessed by high-throughput 16S rRNA amplicon sequencing and represented at the OTU level. The reactors, fed with biomass cultivated in media with replete and low nitrogen content (replete-N BM and low-N BM) were exposed to increasing organic loading rates of 2 and 4 g VS L^-1^ d^-1^ (OLR 2 and OLR 4, respectively). The inoculum and the sampling periods at the end of each OLR were chosen for microbial community monitoring.
